# Psychopathology and Physical Activity as Predictors of Chronic Fatigue Syndrome in the 1958 British Birth Cohort: A Replication Study of the 1946 and 1970 Birth Cohorts

**DOI:** 10.1016/j.annepidem.2010.12.003

**Published:** 2011-05

**Authors:** Laura Goodwin, Peter D. White, Matthew Hotopf, Stephen A. Stansfeld, Charlotte Clark

**Affiliations:** a Centre for Psychiatry, Barts and The London School of Medicine and Dentistry, Queen Mary University of London, London, UK; b King’s College London, Department of Psychological Medicine, Institute of Psychiatry, London, UK

**Keywords:** Chronic Fatigue Syndrome, 1958 British Birth Cohort, Psychopathology, Physical Activity, Etiology, Prospective Cohort Study, CFS/ME, chronic fatigue syndrome/myalgic encephalomyelitis

## Abstract

**Purpose:**

In this study, we investigate whether prospective associations between psychopathology, physical activity, and chronic fatigue syndrome/myalgic encephalomyelitis (CFS/ME) observed in the 1946 and 1970 birth cohorts were replicable in the 1958 British birth cohort.

**Methods:**

Prospective study using the 1958 British birth cohort, which included 98.7% of births from 1 week in March 1958 in England, Wales, and Scotland. The outcome was self-reported CFS/ME by the age of 42 years, at which point 11,419 participants remained in the study. Psychopathology was assessed by the Rutter scales in childhood and the Malaise Inventory in adulthood. Physical activity was reported by the cohort member, mother and teacher in childhood and adulthood.

**Results:**

The prevalence of CFS/ME was 1.0% (95% confidence interval [CI] = 0.9–1.3) and the median age of onset was 34 years. Premorbid psychopathology at 23 years (odds ratio [OR] = 1.85, 95% CI = 1.06–3.22) and 33 years (OR = 2.81, 95% CI = 1.28–6.18) significantly increased the odds of developing CFS/ME, supporting the 1946 cohort findings. Childhood psychopathology, sedentary behavior in childhood, and persistent exercise in adulthood were not associated with CFS/ME.

**Conclusions:**

In cohort studies premorbid psychopathology in adulthood is a replicated risk marker for CFS/ME, whereas premorbid extremes of physical activity are not.

## Introduction

Chronic fatigue syndrome (CFS), also known as myalgic encephalomyelitis (ME) in the United Kingdom, is a syndrome that is primarily characterized by disabling postexertional fatigue, in addition to other symptoms such as musculoskeletal pain and sleep disturbance (1). The etiology of CFS/ME is not established, but there is some evidence linking the condition with premorbid psychopathology (2–4), and premorbid above average physical activity (5–7). A limitation of these findings is that most studies were cross-sectional and it is difficult to determine whether these associations reflect true premorbid risks or result from retrospective response bias. The direction of causality between premorbid levels of activity and psychopathology with CFS/ME can be more unequivocally determined in prospective studies.

Although the majority of studies in the United Kingdom have examined small samples of primary care CFS/ME patients, prospective cohort data has greater validity for examining etiology. Two large scale, nationally representative, birth cohort studies have examined premorbid risk markers for CFS/ME, using the 1946 birth cohort data (8, 9), and the 1970 British cohort data (10). Both of these studies used self-reported diagnoses of CFS/ME, by the age of 53 years in the 1946 cohort, with a focus on early to mid-adulthood risk markers, and by the age of 30 years in the 1970 cohort, with a focus on childhood risk markers. The 1946 cohort analyses found that psychopathology in early adulthood (aged 15–32) was associated with a later diagnosis of CFS/ME (8), whereas in the 1970 cohort childhood psychopathology at age 16 did not predict CFS/ME (10). The studies also found differing evidence for physical activity: the 1946 cohort participants who reported being persistently active at the ages of 13 and 43 years had an increased risk of later CFS/ME (9). By contrast, the 1970 cohort found that taking part in sport in one’s spare time at 10 years was associated with a decreased risk of CFS/ME (10). This disparity between the 1946 and 1970 cohorts could be explained by the age that CFS/ME was reported in the different cohorts, 53 and 30 years respectively, or the different activity measures that were available within the cohorts, but this disparity needs further exploration.

The aims of this replication study were to investigate whether psychopathology and extremes of physical activity predicted CFS/ME in the 1958 British birth cohort. We hypothesized that:

1. Psychopathology in early- to mid-adulthood would predict later CFS/ME at 42 years, in support of the 1946 cohort findings.
2. Sedentary behavior in childhood would be a risk marker for CFS/ME at 42 years, in correspondence with the 1970 cohort findings.
3. Persistent physical activity in early- to mid-adulthood would predict later CFS/ME at 42 years, in support of the 1946 cohort findings.

## Methods

### Sample

The data were taken from the National Child Development Study that is a nationally representative birth cohort study that included 98.7% of births in 1 week in March 1958 (*N* = 17,415) in England, Wales, and Scotland (11). Childhood data was obtained from the mother, teacher, and participant at 7, 11, and 16 years. Adulthood data was gathered at 23, 33, and 42 years. Ninety-two percent of the eligible sample participated at age 7, 92% at age 11, 87% at age 16, 76% at age 23, 71% at age 33, and 71% at age 42 (11, 12).

### Outcome Measures

#### CFS/ME Outcome

Self-report diagnoses of CFS/ME were available at 42 years using an item measuring long-standing health conditions: “Have you ever had CFS/ME?” Participants who answered “yes” to having CFS/ME also reported the age of onset of the condition.

#### Healthy Comparison Group

A representative healthy comparison group (*N* = 9259) was derived, which excluded any self reported cases of CFS/ME, and other functional syndromes or CFS/ME medical exclusions (e.g., irritable bowel syndrome, inflammatory bowel disease). Participants who were likely to meet the other CDC exclusion criteria for CFS/ME (13) were also excluded, including: self reported anorexia/bulimia at 42 years, alcohol misuse (14), substance abuse, and body mass index (BMI) greater than 40. This comparison group was used as a reference category to the CFS/ME group in all of the logistic regression analyses.

### Predictor Measures

The predictor measures were derived to replicate the measures used in the 1946 and 1970 cohorts (10).

#### Psychopathology

Internalizing problems and externalizing problems at age 16 were measured using the teacher version of the Rutter scales (15, 16). The scales were previously found to demonstrate validity, reliability and sensitivity in large populations (16). A score in the top 13% defined a case, the lowest 50% were not a case and the remainder were borderline (17). The internal consistency of these scales has been demonstrated in this cohort (18).

Psychopathology at ages 23, 33, and 42 years was measured using the Malaise Inventory (19), a 24-item scale that assesses symptoms of depression, anxiety, and psychosomatic illness. In this study, a 17-item version of this measure was used, after excluding the seven somatic symptoms that could overlap with the outcome variable of CFS/ME. A cut-off of greater than or equal to seven has been used previously to indicate a high level of psychological distress (20), which represents 29% of the total score of 24 items, therefore an equivalent cut off of greater than or equal to five was used in this study after the exclusion of seven items, which also represents 29% of the total score of 17 items. Examples of items that were excluded from the scale include: “do you feel tired most of the time?,” “Do you often have bad headaches?” The internal consistency for the amended scale in this sample was 0.75, compared with 0.83 for the 24-item scale. A cumulative variable was derived to measure the chronicity of psychopathology between 16 to 33 years (0–3 reports).

#### Measures of Physical Activity

The child’s mother reported on the energy levels of the child at 7 years and the amount of sport played in their spare time at 11 years; teachers rated the child’s ability at sport at 16 years. The “never exercise” and “inactive” categories were used as the reference groups.

In adulthood, physical activity was self-reported at 23 and 33 years. At 23 years, frequency of sports or keep fit activities in an average month was reported, which was dichotomized into participants performing exercise less, or more, than weekly with the former the reference category. At 33 years, participants were asked if they regularly took part in sporting activity, which was categorized as those who did not perform sporting activity, those who participated less than once a week and those who participated at least once a week. Physical activity at work was categorized as participants who performed: no activity at work, some activity, and a high level of activity at work. A combined measure at 33 years classified participants as “inactive” if they performed no exercise at work and no regular sporting activity (the reference group), “less active” if they performed some exercise in either one or both of the categories, “more active” if they performed a high level of activity in one of the categories, and “very active” if they performed a high level of activity in both categories (9). A measure of “persistent” exercise was derived using the measures of sporting activity at 23 and 33 years (9); participants who performed sport at least once a week at both ages were classified as persistent exercisers and were compared with the remaining sample.

### Confounding Measures

#### Social Class and Education

Father’s social class at 7 years, and adult social class at 42 years, was based on current or most recent occupation and categorized using the British Registrar General Classification (21). Educational attainment was measured by qualifications achieved by 33 years.

### Statistical Analysis

Multivariable logistic regression analyses were conducted that examined the associations between the premorbid measures of psychopathology and physical activity and the CFS/ME measure. The statistical analysis replicated the analyses conducted in the 1946 and 1970 cohort studies. All of the analyses were adjusted for age of onset of CFS/ME to ensure that the predictor measures always preceded the onset of the condition.

1. The first analysis examined the univariate associations between the separate measures of psychopathology in childhood and adulthood and CFS/ME, adjusted for gender (8).
2. The second analysis examined the association between sports activity at 11 years, psychopathology at 42 years, and CFS/ME in a multivariable model, including the variables gender, father’s social class, social class at 42 years, and childhood chronic illness at 11 years. This replicated the multivariable model tested in the 1970 cohort (10).
3. The final analysis examined the univariate associations between childhood and adulthood measures of physical activity and CFS/ME, adjusting for gender, father’s social class, social class at 42 years, and highest level of qualification at 33 years (9).

#### Missing Data

Multiple imputation was used to address the issue of missing data on the analyses, using the ICE program in STATA (Version 10). The imputation equation included all of the variables that were included in the final analyses, which is recommended as best practice for multiple imputation (22): these were the CFS/ME outcome measures, and measures of psychopathology and physical activity, and the demographic measures. Further variables that predict attrition were also included: these were gender, socioeconomic position at 7 and 42, and qualifications at 33 (23). The proportion of data missing on the dependent variable was 0.4%. Missing data on the independent variables ranged from less than 1% to 16% except for psychopathology at 16 years (25%), aptitude for sports at 16 years rated by teacher (25%), and physical activity in job at 33 years (30%). All living participants were included in the imputation, but analyses in this study were restricted to those who participated in the 42 year study. Five cycles of the imputation were run and analyses indicated that the measures were stable across the imputations. Parameter estimates from the five imputations were estimated using the MIM function in STATA. The ORs presented in the results tables use imputed data, but analyses using the imputed and nonimputed data showed a similar pattern of results.

## Results

The unimputed prevalence of CFS/ME in this cohort was 1.0% (95% CI = 0.9–1.3), with 123 participants (50 males and 73 females) reporting the condition. The median reported age of onset for CFS/ME was 34 years (mean = 32, standard deviation [SD] = 7). This compared with a prevalence of 1.1% (95% CI = 0.8–1.5) in the 1946 cohort and 0.8% (95% CI = 0.7–1.0) in the 1970 cohort.

### Psychopathology in Early- to Mid-Adulthood

Table 1 shows that psychopathology at 16 years was not associated with CFS/ME reported at 42 years. Psychopathology in early adulthood, at 23 and 33 years, was significantly associated with 1.9- and 2.8-fold increases in odds for CFS/ME, respectively, in support of the 1946 cohort findings. Cumulatively, one report of psychopathology in adulthood was not associated with increase in odds of CFS/ME, but two or more reports were significantly associated with a 3.2-fold increase in odds. Table 2 shows that there was also evidence across the cohorts for a cross-sectional association between CFS/ME and psychopathology.

### Sedentary Behavior in Childhood

Table 3 shows that the energy level of the child at 7 years, reported by the mother, was not associated with CFS/ME. This did not support the 1946 cohort that found that high levels of energy at 13 years reported by the teacher were associated with an increase in odds of CFS/ME. Table 4 shows that there was no evidence that playing sports in spare time at age 11 years was associated with CFS/ME, providing no support for the 1970 cohort findings.

### Physical Activity in Early- to Mid-Adulthood

Table 3 shows that physical activity at 23 and 33 years were not associated with CFS/ME. Furthermore, there was no evidence that persistent exercisers at 23 and 33 years had either a decrease or increase in the odds of CFS/ME.

## Discussion

This study examined psychopathology and self-reported physical activity as predictors of CFS/ME in the 1958 cohort, and compared the findings with the 1946 and 1970 cohorts. The current data supported the previous finding that psychopathology in early- and mid-adulthood predicted later CFS/ME (8). There was also support across the cohorts for a cross-sectional association between CFS/ME and psychopathology (10), in agreement with previous research (2–4, 24, 25). In contrast, the data did not support previous findings regarding extremes of physical activity. There was no evidence that performing sporting activity at age 11 was associated with reduced odds of CFS/ME (10), or that persistent physical activity in early- to mid-adulthood increased the risk of CFS/ME (9). This study provided no evidence for an association between physical activity, in either childhood or adulthood, and CFS/ME.

### Possible Explanations for Disparities Between the Cohorts

This replication study aimed to conduct the same data analyses as the previous cohort studies, and whilst the findings on psychopathology were stable across the cohorts, there were major cohort differences with regard to the potential association between physical activity and CFS/ME. The current study suggests that there is no association between CFS/ME and extremes of physical activity, whereas the 1946 cohort findings showed that persistent activity in adulthood increased the odds of CFS/ME (9) and the 1970 cohort findings suggested that sedentary children had a greater risk of CFS/ME in adulthood (10). These contrasting findings may result from either a lack of sensitivity in the measurement of physical activity, or from cohort variability in regard to patterns of physical activity. The rates of physical activity presented in tables 3 and 4 indicate that levels of physical activity increased over each consecutive cohort. The proportion of participants who performed persistent physical activity at least once a week in adulthood in the 1946 cohort was 7.9% compared to 25.0% in the 1958 cohort. Furthermore, the frequency of sports played “most days” or “often” at 11 years increased from 45.7% in the 1958 cohort, to 54.0% in the 1970 cohort. There is also some empirical evidence to support a trend of increasing levels of activity over time (26). One potential explanation for the disparity in findings for physical activity may be the cohort differences in level of activity; the proportion of the sample included in the reference category of being “inactive” differs quite considerably across the different cohorts, therefore giving rise to the possibility of potentially significant differences in the characteristics of the cohort members defined as inactive in each cohort.

A further implication of these cohort data concerns the subjective nature of the measures of physical activity; reported physical activity is not always associated with objective measures of activity or physical fitness (27) that may show a stronger association with CFS/ME (28). In all of the cohorts, physical activity was measured using single items at each time point that may not reflect variability in the time between data collections nor accurately capture the complexity of physical activity behaviors. Analyses of the 1958 cohort showed that the pattern of physical activity from childhood to 42 years is relatively unstable (29, 30), suggesting that either these self-report items are not accurate or that individuals show wide variation in their exercise patterns across the lifespan. Measurement of physical activity was developed in the 1990s to include a more specific measure of duration and frequency of different types of exercise to calculate energy expenditure (27) in line with current government recommendations for activity (30). This data is available for the 1958 cohort at age 45, and this approach should be promoted for future research. Retrospective reports of physical activity in CFS/ME patients suggest that this group may exhibit high levels of physical activity before onset (31), but the current study provides no support for this finding in a prospective population study, within the constraints of the measures that are available. There are potential issues with the capability of the cohort studies to assess what may be a temporally influenced association between physical activity and CFS/ME; physical activity was only reported every 10 years at the main data collections, and it is possible that premorbid overactivity nearer to the time of onset, which we have not been able to characterize in this data, could be a risk marker.

### Strengths and Limitations

There are a number of strengths to this prospective cohort study. This design allowed us to examine risk and preventive markers for CFS/ME measured before the onset of CFS/ME, compared to previous cross-sectional studies using retrospectively gathered data. The 1958 cohort also has more participants than the 1946 cohort and, furthermore, in the current study missing data was addressed using multiple imputation. This enabled us to deal with the potential attrition bias, which the previous cohort analyses do not, whilst increasing the power of the data further. Finally, the benefit of comparing across the cohorts is that any consistent risk or preventive markers can be concluded as being stable markers for CFS/ME, but if there is mixed evidence across the cohorts then this highlights an area requiring further investigation.

The main limitation of this study is the difference between the three cohort studies in the measures used, and the potential cultural and social differences between individuals born in the different eras. There may also be differences in risk factors for CFS/ME between individuals who develop CFS/ME in early-adulthood compared to those who develop CFS/ME in mid- to late-adulthood that cannot be identified in this replication study. A further limitation is the reliance on a self-report of CFS/ME. However, the outcome was also self-report in the 1946 and 1970 cohorts so the outcome measurement should not have resulted in the cohort differences observed. As with any self-report of health, there remains a potential for misdiagnosis, but the prevalence rates reported in this data are very similar to prevalence estimates in England (32), suggesting that there was not a high number of participants reporting a diagnosis that they did not have. Finally, there are weaknesses with the self-report nature of these national cohort studies, and possible issues of social desirability, which is an unavoidable weakness for a data set of this size and power.

### Implications of This Study

This study provides strong evidence that adult psychopathology is a premorbid risk marker for CFS/ME. The next step is to better understand this risk. CFS/ME patients are also more likely than controls to experience psychological distress after the onset of the condition, and treatment of this comorbid psychopathology will improve the health of patients. Inactivity and overactivity were not found to be significant risk markers for CFS/ME, and this study has highlighted issues with the assessment of self-reported physical activity in population studies.

### Conclusions and Future Research

This study has found that premorbid psychopathology in adulthood is a risk marker for CFS/ME across the cohort studies. An association between premorbid physical activity and CFS/ME has not been confirmed. This study provides evidence that research findings across the birth cohorts can differ and that more cross-cohort comparison studies are needed to strengthen the evidence for possible risk markers.

## Figures and Tables

**Table 1:** Replication of the 1946 cohort showing psychopathology predicting CFS/ME

Cohort	Age measured (yr)	Predictor measures	Unimputedfrequencies *N* (%)	Imputed frequencies *N* (%)	Sub-sample with CFS/ME (%)	OR (95% CI)	*p*
1946∗	15–32	Psychiatric disorder					
		None	1673 (57.8)	—	0.96	1.00	
		Mild/moderate	1040 (35.9)	—	1.15	1.22 (0.57–2.59)	
		Severe	181 (6.3)	—	3.31	2.97 (1.13–7.78)	0.06 (trend)
	36	Psychiatric disorder (using present state examination)					
		No	2503 (94.1)	—	1.00	1.00	
		Yes	156 (5.9)	—	3.85	3.47 (1.39–8.66)	0.008
	43	Psychiatric disorder (using Psychiatric Symptom Frequency scale)					
		No	1968 (71.0)	—	0.76	1.00	
		Yes	803 (29.0)	—	1.50	1.80 (0.84–3.89)	0.13
1958∗	16	Psychopathology (any externalizing or internalizing problems)					
		No	6536 (76.4)	8581 (75.1)	1.10	1.00	
		Yes	2016 (23.6)	2838 (24.9)	1.16	1.12 (0.73–1.72)	0.60
	23	Psychopathology (using Malaise Inventory)					
		No	8572 (89.0)	10,109 (88.5)	1.04	1.00	
		Yes	1061 (11.0)	1310 (11.5)	1.68	1.85 (1.06–3.22)	0.03
	33	Psychopathology (using Malaise Inventory)					
		No	9001 (91.8)	10,425 (91.3)	0.99	1.00	
		Yes	806 (8.2)	994 (8.7)	2.41	2.81 (1.28–6.18)	0.01
	16–33	Any psychopathology					
		None	4528 (68.7)	7459 (65.3)	0.98	1.00	
		1 report	1576 (23.9)	2994 (26.2)	1.10	1.01 (0.47–2.16)	0.98
		2–3 reports	485 (7.4)	965 (8.5)	2.18	3.23 (1.27–8.19)	0.02

CFS/ME = chronic fatigue syndrome/myalgic encephalomyelitis; CI = confidence interval; OR = odds ratio.

∗ The analyses were adjusted for gender.

**Table 2:** Replication of the 1970 cohort showing psychopathology predicting CFS/ME

Cohort	Age measured	Predictor measures	Unimputed frequencies *N* (%)	Imputed frequencies *N* (%)	Sub-sample with CFS/ME (%)	OR (95% CI)	*p*
1958*	42	High scorer on malaise inventory					
		No	9696 (84.9)	9696 (84.9)	0.79	1.00	
		Yes	1723 (15.1)	1723 (15.1)	2.90	5.56 (3.15–9.82)	<0.001
1970†	30	High scorer on malaise inventory					
		No	9459 (84.0)	—	0.58‡	1.00	
		Yes	1802 (16.0)	—	2.11‡	2.6 (1.6 to 4.3)	<0.001

CFS/ME = chronic fatigue syndrome/myalgic encephalomyelitis; CI = confidence interval; OR = odds ratio.

∗ The analysis was adjusted for gender, father’s social class, participants’ social class, presence of long-standing medical condition at 11 years, and sport played in spare time at 11 years.

† The analysis was adjusted for gender, father’s social class, participants’ social class, mother’s qualifications, presence of long-standing medical condition at 10 years, and sport played in spare time at 10 years.

‡ The percentages are estimated from the frequencies available.

**Table 3:** Replication of the 1946 cohort showing physical activity predicting CFS/ME

Cohort	Age measured	Predictor measures	Unimputedfrequencies *N* (%)	Imputed frequencies *N* (%)	Sub-sample with CFS/ME (%)	OR (95% CI)	*p*
1946*	13	Energy levels (reported by teacher)					
		Always tired	111 (4.4)	—	0.00	N/A	
		Normal	2227 (88.0)	—	0.99	1.00	
		Extreme	192 (7.6)	—	3.13	3.58 (1.29–9.93)	0.006
		Ability at sport (reported by teacher)					
		Below average	355 (14.1)	—	0.28	1.00	
		Average	1702 (67.7)	—	1.12	2.79 (0.37–21.26)	
		Above average	458 (18.2)	—	1.75	3.40 (0.39–29.46)	0.29
	31	Sports or keep fit activities					
		<Weekly	1120 (86.0)	—	0.89	1.00	
		>Weekly	182 (14.0)	—	2.75	3.52 (0.99–12.05)	0.05
	36	Physical activity (sports, cycling, walking, work, heavy gardening, or DIY)					
		Inactive	183 (6.9)	—	1.09	1.00	
		Less active	1183 (44.9)	—	0.85	1.24 (0.15–10.04)	
		More active	820 (31.1)	—	1.10	1.76 (0.22–14.26)	
		Very active	451 (17.1)	—	2.22	3.17 (0.39–25.71)	0.06
	31–43	Persistent or vigorous physical activity at least once a week					
		No	1197 (92.1)	—	0.67	1.00	
		Yes	102 (7.9)	—	3.92	10.80 (2.66–43.79)	0.001
1958*	7	Energy levels (reported by mother)					
		Inactive	375 (3.8)	432 (3.8)	1.39	1.00	
		Normally active	8148 (81.8)	9328 (81.7)	1.09	0.71 (0.31–1.64)	0.43
		Overactive	1441 (14.5)	1659 (14.5)	1.15	0.73 (0.27–1.96)	0.53
	16	Ability at sport (reported by teacher)					
		Below average	1557 (18.2)	2107 (18.5)	1.47	1.00	
		Average	4791 (56.0)	6374 (55.8)	0.99	0.68 (0.38–1.19)	0.17
		Above average	2213 (25.8)	2938 (25.7)	1.12	0.79 (0.42–1.48)	0.45
	23	Sports or keep fit activities					
		<Weekly	6569 (68.1)	7764 (68.0)	1.18	1.00	
		>Weekly	3081 (31.9)	3655 (32.0)	0.96	0.84 (0.53–1.31)	0.43
	33	Physical activity (combined measure of sports activities and activity in job)					
		Inactive	339 (4.3)	495 (4.3)	1.45	1.00	
		Less active	1309 (16.4)	1859 (16.3)	1.38	2.04 (0.24–17.07)	0.47
		More active	4718 (59.3)	6707 (58.7)	0.98	2.47 (0.33–18.34)	0.36
		Very active	1591 (20.0)	2358 (20.6)	1.20	2.95 (0.38–23.07)	0.29
	23 and 33	Persistent physical activity at least once a week					
		No	6387 (74.8)	8568 (75.0)	1.19	1.00	
		Yes	2151 (25.2)	2851 (25.0)	0.86	1.12 (0.57–2.19)	0.83

CFS/ME = chronic fatigue syndrome/myalgic encephalomyelitis; CI = confidence interval; OR = odds ratio.

∗ The analyses were adjusted for gender, father’s social class, participants’ social class, and highest level of qualification.

**Table 4:** Replication of the 1970 cohort showing physical activity predicting CFS/ME

Cohort	Age measured	Predictor measures	Unimputed frequencies *N* (%)	Imputed frequencies *N* (%)	Sub-sample with CFS/ME (%)	OR (95% CI)	*p*
1958∗	11	Sport played in spare time					
		Hardly ever	1074 (11.3)	1316 (11.5)	1.29	1.00	
		Sometimes	4082 (43.0)	4907 (43.0)	1.08	0.96 (0.41–2.27)	0.93
		Most days	4347 (45.7)	5195 (45.5)	1.10	0.85 (0.35–2.06)	0.72
1970†	10	Sport played in spare time					
		Never or hardly ever	775 (8.0)	—	1.94‡	1.00	
		Sometimes	3780 (39.0)	—	1.03‡	0.5 (0.3– 1.0)	
		Often	5234 (54.0)	—	0.75‡	0.5 (0.2–0.9)	0.04 for trend

CFS/ME = chronic fatigue syndrome/myalgic encephalomyelitis; CI = confidence interval; OR = odds ratio.

∗ The analysis was adjusted for gender, father’s social class, participants’ social class, presence of long-standing medical condition at 11 years, and psychopathology at 42 years.

† The analysis was adjusted for gender, father’s social class, participants’ social class, mother’s qualifications, presence of long-standing medical condition at 10 years, and psychopathology at 30 years.

‡ The percentages are estimated from the frequencies available.
